# Normal reference values for magnetic resonance imaging measurements of the fetal internal jugular veins in middle and late pregnancy

**DOI:** 10.1007/s00247-023-05594-w

**Published:** 2023-03-28

**Authors:** Duo Gao, Xin Liu, Yimin Cao, Zexi Yi, Xiaobin Gao, Ying Li, Cuili Cao, Zuojun Geng, Lixia Zhou

**Affiliations:** 1 Department of Medical Imaging, The Second Hospital of Hebei Medical University, Hebei Province, 215 Heping Western Road, Shijiazhuang, 050000 China; 2 Department of Anatomy, School of Basic Medical Sciences, Hebei Medical University, Hebei Province, 361 Zhongshan Eastern Road, Shijiazhuang, 050017 China

**Keywords:** Aneurysm, Cross-sectional area, Fetus, Internal jugular vein, Magnetic resonance imaging, Vein of Galen

## Abstract

**Background:**

At present, there is a lack of normal magnetic resonance imaging (MRI) morphometric reference values for fetal internal jugular veins during middle and late pregnancy.

**Objective:**

We used MRI to assess the morphology and cross-sectional area of the internal jugular veins of fetuses during middle and late pregnancy and to explore the clinical value of these parameters.

**Materials and methods:**

The MRI images of 126 fetuses in middle and late pregnancy were retrospectively analysed to determine the optimal sequence for imaging the internal jugular veins. Morphological observation of the fetal internal jugular veins in each gestational week was carried out, lumen cross-sectional area was measured and the relationship between these data and gestational age was analysed.

**Results:**

The balanced steady-state free precession sequence was superior to other MRI sequences used for fetal imaging. The cross section of fetal internal jugular veins was predominantly circular in both the middle and late stages of pregnancy, however the prevalence of an oval cross section was significantly higher in the late gestational age group. The cross-sectional area of the lumen of the fetal internal jugular veins increased with increasing gestational age. Fetal jugular vein asymmetry was common, with the right jugular vein being dominant in the high gestational age group.

**Conclusion:**

We provide normal reference values for fetal internal jugular veins measured by MRI. These values may form the basis for clinical assessment of abnormal dilation or stenosis.

**Supplementary Information:**

The online version contains supplementary material available at 10.1007/s00247-023-05594-w.

## Introduction

In recent years, fetal magnetic resonance imaging (MRI) technology has developed rapidly; 1.5-tesla (T) MRI is widely used. Literature reports on fetal MRI are increasing year on year. Fetal organ systems develop rapidly and the diagnosis of many fetal diseases and malformations requires reference to normative data for the corresponding gestational age. At present, there are still only a few reports on MRI parameters of normal fetal structures [1] and fetal MRI diagnosis often requires ultrasound data as a reference.

The incidence of fetal craniocerebral diseases and malformations is high, second only to that of cardiovascular disease, so measurement data of normal fetal craniocerebral structures are urgently needed [2]. Brain tissue is rich in blood supply, and many intracranial diseases affect haemodynamics and cause abnormalities in venous drainage [3, 4]. The vein of Galen and straight sinus are mainly involved in the drainage of the deep cerebral veins. Some scholars have measured the diameters of the vein of Galen and straight sinus in the fetal period and discussed their development pattern [5]. The internal jugular veins are the largest draining veins of the head and neck and they are also the bridge between the brain and the heart. Most of the deep and superficial brain veins empty into the internal jugular veins, the blood drains out of the skull through them and many prenatal diseases can affect the internal jugular veins.

At present, most imaging studies on fetal brain development focus on morphological changes and volume measurement of the brain parenchyma [6]. The majority of studies on the fetal cerebral vessels use ultrasound and they mainly focus on monitoring blood flow in the cerebral arteries [7]. Three-dimensional (D) ultrasound shows that fetal brain volume and blood flow increase with increasing gestational age and venous parameters may be important in brain vascularisation [8].

The internal jugular veins play an important role in the venous return system of the fetus, and many prenatal malformations may affect their blood flow and morphological development. It has been shown  that the internal jugular veins may be involved in diseases of the fetal brain, neck, and cardiovascular system as well as in systemic diseases. Zhou LX et al. [9] and Wagner M et al. [10] reported that the internal jugular veins dilated when the fetuses had a vein of Galen aneurysmal malformation (VGAM), while other authors reported that the internal jugular vein blood flow of fetuses with increased nuchal translucency [11] or intrauterine growth restriction [12] was higher than that of normal fetuses. When fetal oedema is accompanied by ventricular dilation and cervical lymph node enlargement, the internal jugular veins will also present with secondary changes [13]. Dilation or stenosis of the internal jugular veins often occurs in fetuses with congenital heart disease [14, 15].

Although there are currently a few basic and clinical studies on normal fetal internal jugular veins, the sequence of internal jugular vein development and morphological change has not yet been described [1]. In this study, the morphometry of fetal internal jugular veins was observed by MRI in the middle and late stages of pregnancy, providing a reference for the diagnosis and subsequent treatment of fetal diseases.

## Materials and methods

### Participants

A retrospective analysis was performed on the imaging data of 126 singleton fetuses in middle and late pregnancy who underwent MRI examination at the Second Hospital of Hebei Medical University from October 2019 to July 2020. The exclusion  criteria were as follows: (1) pregnant women <18 years old and (2) abnormality found on ultrasound or MR examination.

Some fetuses with abnormal internal jugular veins were further examined by MRI in the Second Hospital of Hebei Medical University from October 2019 to July 2020 to analyse the changes in the internal jugular vein in various conditions such as VGAM, fetal oedema with ventricular dilation and fetal growth retardation.

The flow chart for patient recruitment to this study is shown in Supplementary Material 1.

### Magnetic resonance protocols

Scans were performed on a 1.5-T MRI scanner (Signa, General Electric Company, Grandview Blvd, Waukesha, WI). The scanning sequences and parameters of fetal transverse, coronal and sagittal images were as follows: balanced steady-state free precession (B-SSFP) sequence, TR 5.3 ms, TE 2.3 ms, layer thickness 6 mm; single-shot fast spin echo (SS-FSE) sequence, TR 2,000 ms, TE 140 ms; fast inversion recovery motion (FIRM) inhibition sequence, TR 150 ms, TE 4.2 ms; diffusion-weighted imaging (DWI) sequence, TR 3,274 ms, TE 75 ms, B=0, 700 s/mm^2^, layer thickness 5 mm, field of view 380 mm × 380 mm.

### Image analysis

In accordance with previous fetal MRI sequence studies [16], an evaluation of the display quality of the fetal internal jugular veins of various MRI sequences was conducted. Three radiologists (G.Z.J., Z.L.X. and G.X.B. with 33, 19 and 17 years of experience, respectively) compared the sequences (B-SSFP, SS-FSE, DWI and FIRM) in conensus, to assess the visibility of the internal jugular veins and to determine the optimal sequence. The cross-sectional shape of the internal jugular veins in the middle and late pregnancy groups was recorded. Left and right fetal internal jugular veins were independently measured in cross section by two radiologists (G.D. and L.X. with 7 and 4 years of experience, respectively) at the level of the atlantoaxial joint. Each vein was measured three times and the average values taken.

### Statistical analysis

Statistical analysis was performed using the SPSS 27.0 (IBM, Chicago, IL) and GraphPad Prism 9.0.0 (GraphPad Software, Inc., La Jolla, CA). Measurement data is expressed as mean ± standard deviation (SD). The cross-sectional area of the internal jugular veins and gestational age were analysed by regression. A paired  *T* test was performed on the cross-sectional area of the left and right internal jugular veins. Fisher’s exact test was used to compare the morphological proportions between the middle- and late- trimester groups. Bland–Altman analysis was used to analyse the interobserver consistency of the data measured by two radiologists. The intraclass correlation coefficient (ICC) was used to determine the intraobserver agreement. The ICC analysis was conducted using a two-way mixed effect model. The ICC values were expressed with a 95% confidence interval (CI). An ICC of ≥ 0.75 was interpreted as excellent agreement [17]. A value of *P* < 0.05 was considered indicative of statistical significance.

## Results

### Imaging findings

**Fig. 1:**
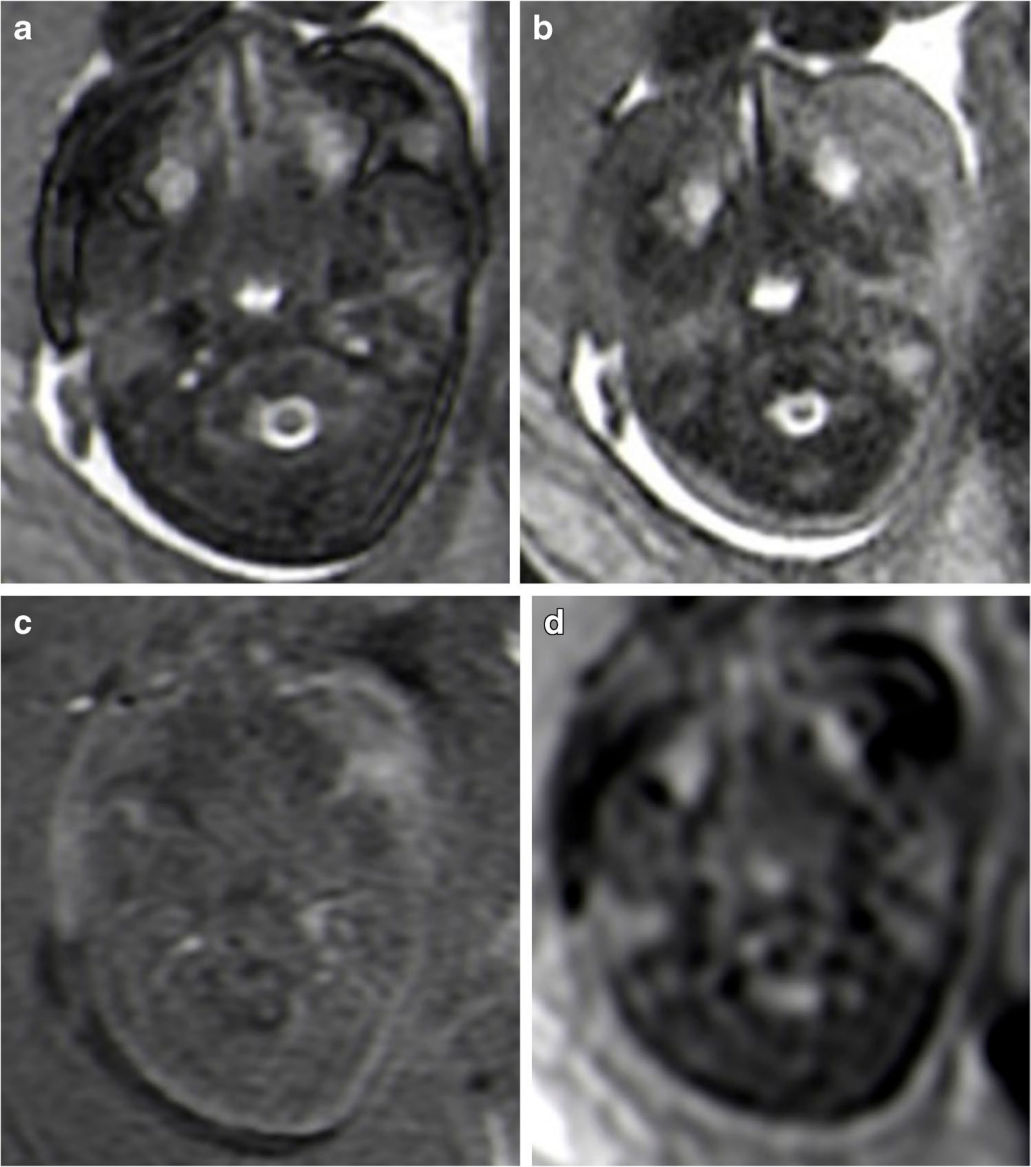
Magnetic resonance (MR) images in a 30-week-old female fetus. **a** Axial balanced steady-state free precession sequence at the level of the atlantoaxial joint, clearly shows the internal jugular veins. **b–d** There is poor visualisation of the internal jugular veins and cervical structures on the axial single shot fast spin echo (**b**), axial fast inversion recovery motion (**c**) and axial diffusion-weighted imaging (**d**) sequences

Axial MR images showed the bilateral internal jugular veins of the same fetus for all sequences (B-SSFP, SS-FSE, FIRM and DWI) (Fig. 1). The fetal bilateral internal jugular veins showed high signal intensity on B-SSFP (higher signal than that of the adjacent internal carotid arteries) and contrasted well against the surrounding low signal soft tissues, hence B-SSFP was considered the optimal sequence. On the SS-FSE, FIRM and DWI sequences, both the internal jugular veins and surrounding tissues showed low signal, making the veins difficult to distinguish. Furthermore, on the DWI sequences the internal jugular veins had a blurred contour.

### General data

A total of 126 pregnant women with a singleton fetus in the middle and late trimester were included in the study. The gestational age was 21–39 weeks. Patients were subclassified into a middle trimester group (21–27 weeks of gestation) and a late trimester group (28–39 weeks of gestation).

Paired *T* test showed that the cross-sectional area of the right internal jugular vein was significantly larger than that of the left internal jugular vein (*P* < 0.05) (Table 1). This difference was apparent from 23 weeks of gestation.

**Table 1:** Cross-sectional area of internal jugular veins at different gestational ages

Gestational age	Number	Cross-sectional area (mean ± SD, mm^2^)		
		Left	Right	*P*
21 ~ 22	7	5.85 ± 0.90	6.15 ± 1.13	0.17
23 ~ 24	14	5.86 ± 1.05	6.85 ± 1.92	0.0006
25 ~ 26	8	8.02 ± 1.06	8.38 ± 1.05	0.037
27 ~ 28	13	8.82 ± 1.08	9.80 ± 1.41	< 0.0001
29 ~ 30	18	8.62 ± 1.20	10.71 ± 1.37	< 0.0001
31 ~ 32	30	10.43 ± 1.64	11.69 ± 1.41	< 0.0001
33 ~ 34	15	11.05 ± 1.89	12.90 ± 1.72	< 0.0001
35 ~ 36	13	13.58 ± 2.70	14.83 ± 2.75	0.036
37 ~ 38	7	12.37 ± 2.31	13.53 ± 3.27	0.21
39	1	13.33 ± 0.58	15.33 ± 1.53	0.22

### Correlation analysis between the cross-sectional area of the internal jugular veins and gestational age

**Fig. 2:**
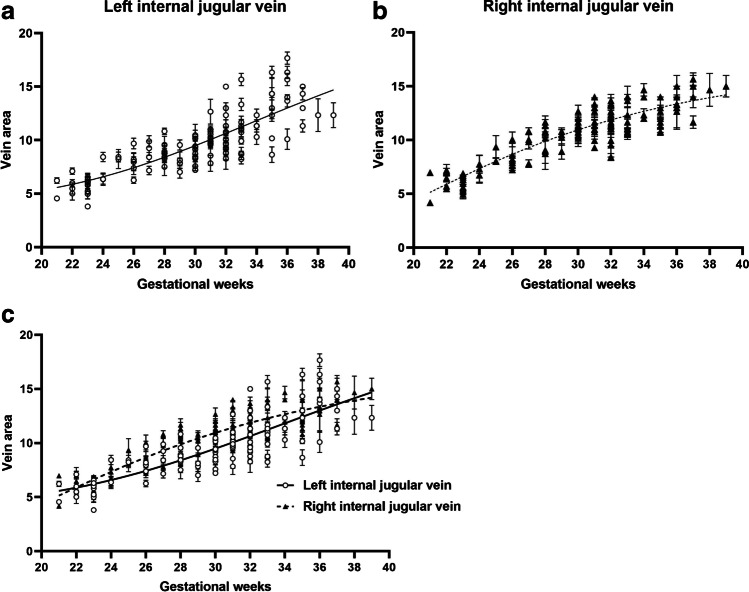
Correlation of cross-sectional area of bilateral internal jugular veins with gestational age. **a** Correlation between left internal jugular vein and gestational age. **b** Correlation between right internal jugular vein and gestational age. **c** Comparison of correlation curves between left and right internal jugular veins and gestational age

**Fig. 3:**
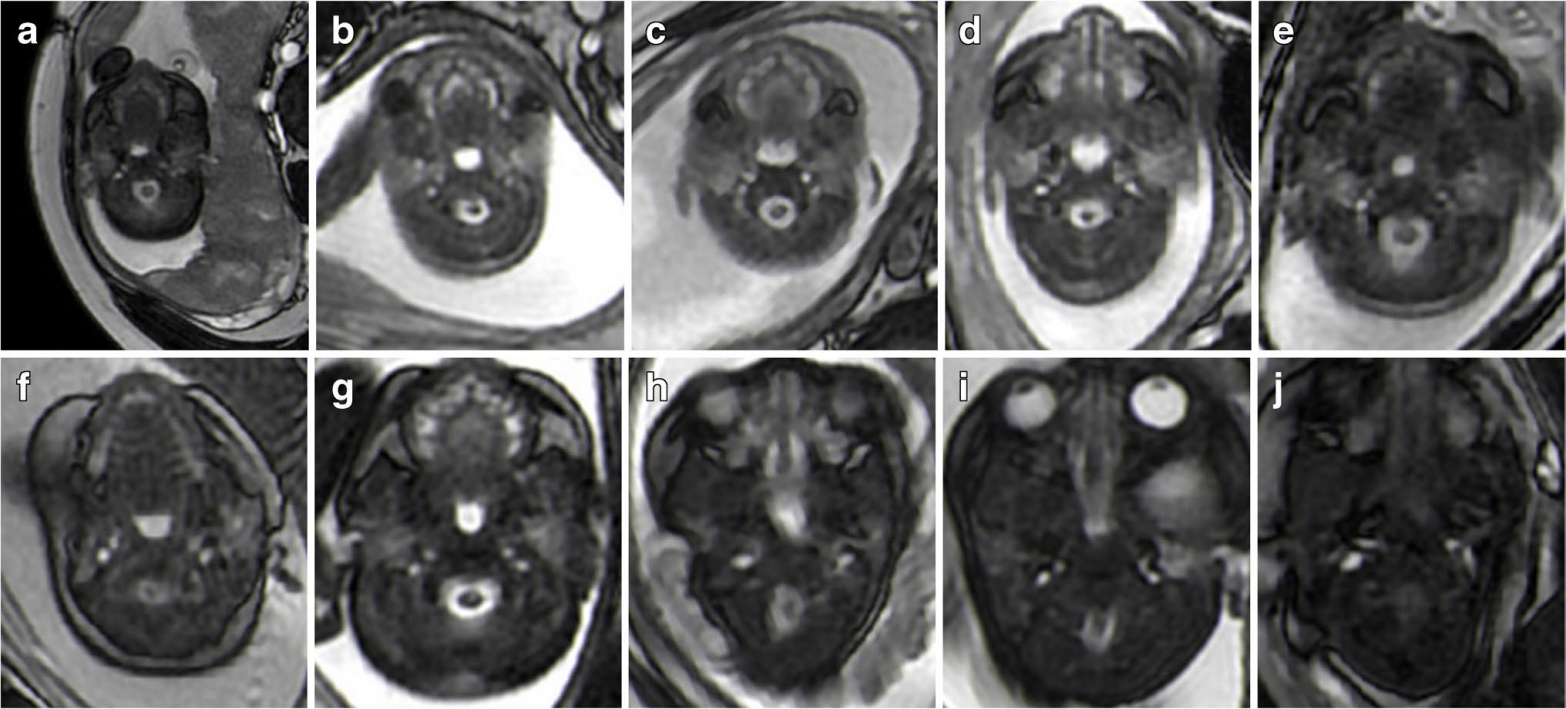
Axial balanced steady-state free precession magnetic resonance images of bilateral internal jugular veins of fetuses from 21–39 weeks of gestation. **a** 21-week-old female fetus. **b** 22-week-old male fetus. **c** 24-week-old male fetus **d** 28-week-old male fetus **e** 30-week-old female fetus **f** 32-week-old male fetus **g** 34-week-old female fetus **h** 36-week-old male fetus **i** 38-week-old male fetus **j** 39-week-old male fetus

**Fig. 4:**
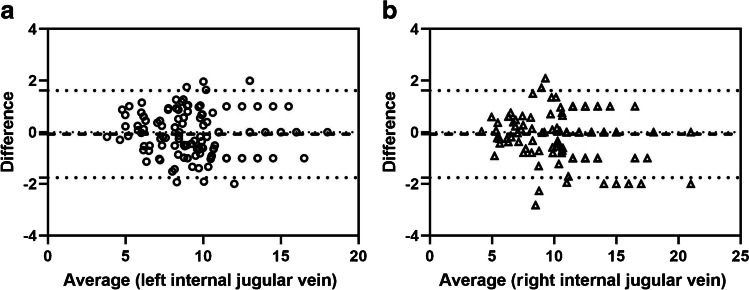
Bland–Altman plots of difference vs the average cross-sectional area of left (**a**) and right (**b**) internal jugular veins. Measurement of the cross-sectional area of the bilateral internal jugular veins was consistent between two radiologists

The change in the right internal jugular vein with gestational age was similar to that of the upper part of an S-shaped curve, while the change in the left internal jugular vein with gestational age was similar to that of the  lower part of an S-shaped curve. The cross-sectional area of the right internal jugular vein was larger than that of the left (Fig. 2). With increase in gestational age the shape and size of the internal jugular veins also changed (Fig. 3). The two observers had good consistency (Fig. 4) with only a small number of measurements outside the 95% limits of agreement. The ICCs for intraobserver agreement were 0.94 (0.92 to 0.95, 95% CI) and 0.94 (0.92 to 0.96, 95% CI), respectively.

### Morphological changes in the cross section of the internal jugular veins

**Fig. 5:**
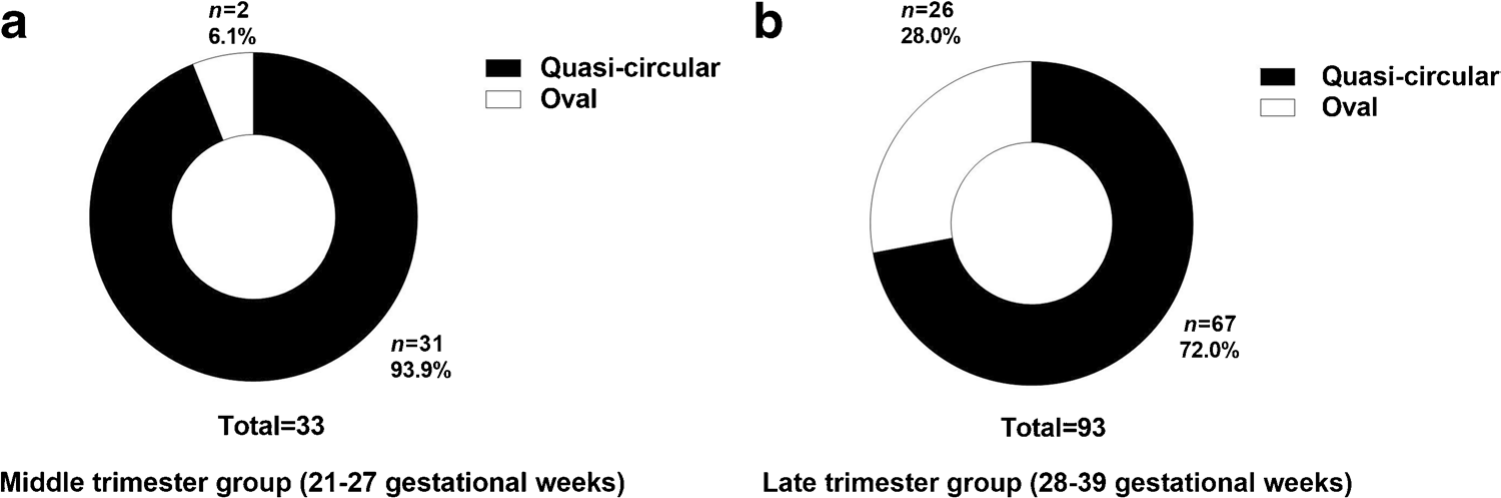
Fetal internal jugular vein morphology in the middle- (**a**) and late- (**b**) trimester groups. **a** Morphological classification of fetal internal jugular veins at 21–27 weeks of gestation show that the proportion of quasi-circular and oval components were 93.9% and 6.1%, respectively; (**b**) morphological classification of fetal internal jugular veins at 28–39 weeks of gestation show that the proportion of quasi-circular and oval components were 72.0% and 28.0%, respectively

The cross section of the internal jugular veins was quasicircular in 77.8% and oval in 22.2%. The proportions of oval in the late and middle gestational age groups were 28.0% and 6.1%, respectively (Fig. 5). There was a significant difference in the elliptical proportions between the late and middle gestational age groups (*P*=0.0077).

### Abnormal internal jugular veins

A total of 28 patients had abnormality, including VGAM in 10 patients, fetal oedema in 10 patients, leptomeningeal arteriovenous fistula in 5 patients and intrauterine fetal growth restriction in 3 patients.

**Fig. 6:**
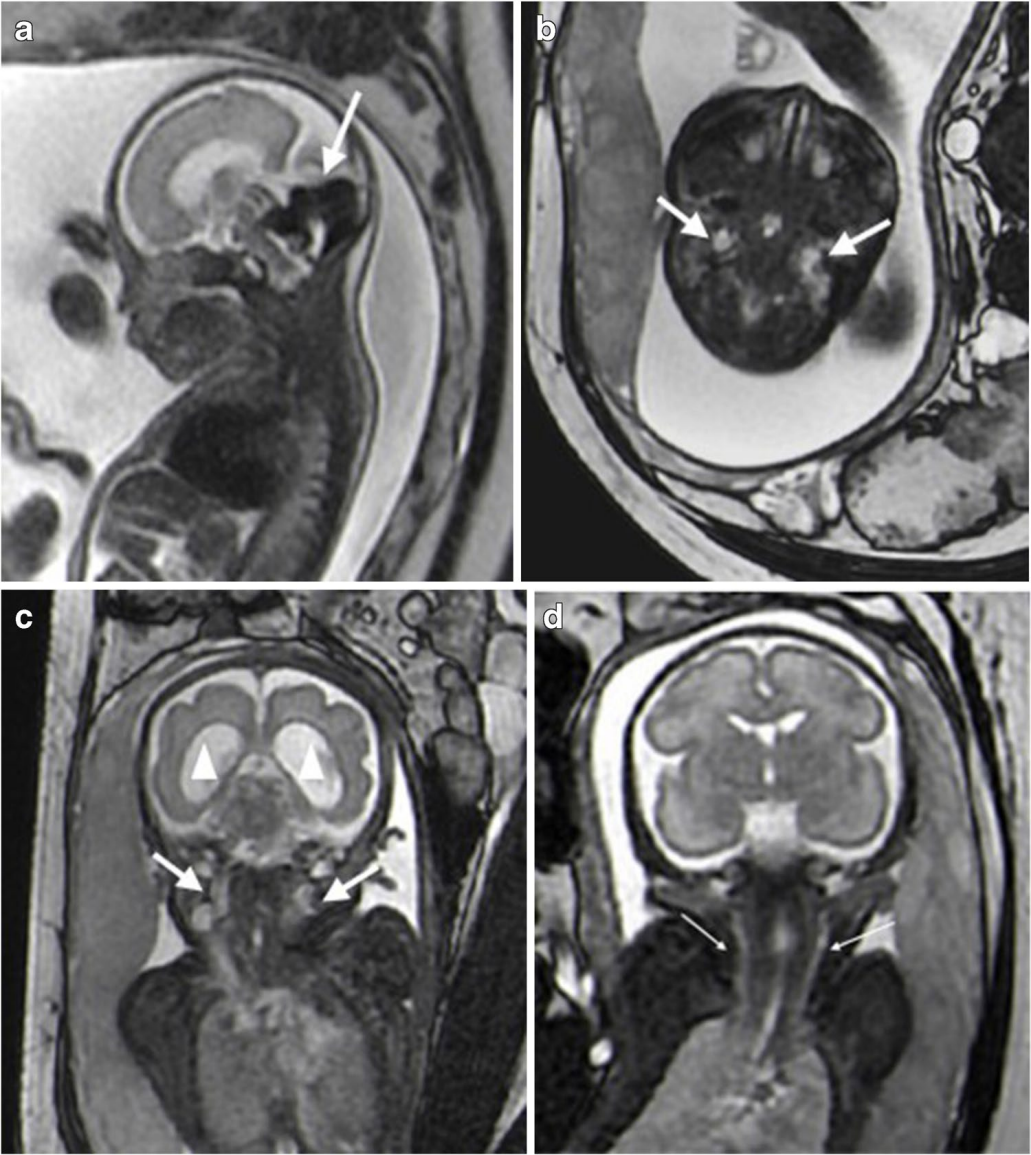
Magnetic resonance imaging in a 28-week-old female fetus with a vein of Galen aneurysm. **a** A sagittal single shot fast spin echo image shows the vein of Galen dilatation (*arrow*). **b** An axial balanced steady-state free precession (B-SSFP) image shows bilateral internal jugular vein dilatation at the level of the atlantoaxial joint (*arrows*). **c** A coronal B-SSFP image shows bilateral internal jugular vein dilatation (*arrows*) and lateral ventricle enlargement (*arrowheads*). **d** A coronal B-SSFP image of a normal fetus at 28 weeks of gestation with normal bilateral internal jugular veins (*arrows*)

A vein of Galen aneurysm was the most common finding in patients with abnormal fetal internal jugular veins. In one patient, ultrasound revealed dilatation of the bilateral brachiocephalic and internal jugular veins with abnormal intracranial blood flow. On MRI, VGAM was found, together with dilation of the bilateral transverse sinuses, sigmoid sinus and internal jugular veins (Fig. 6).

**Fig. 7:**
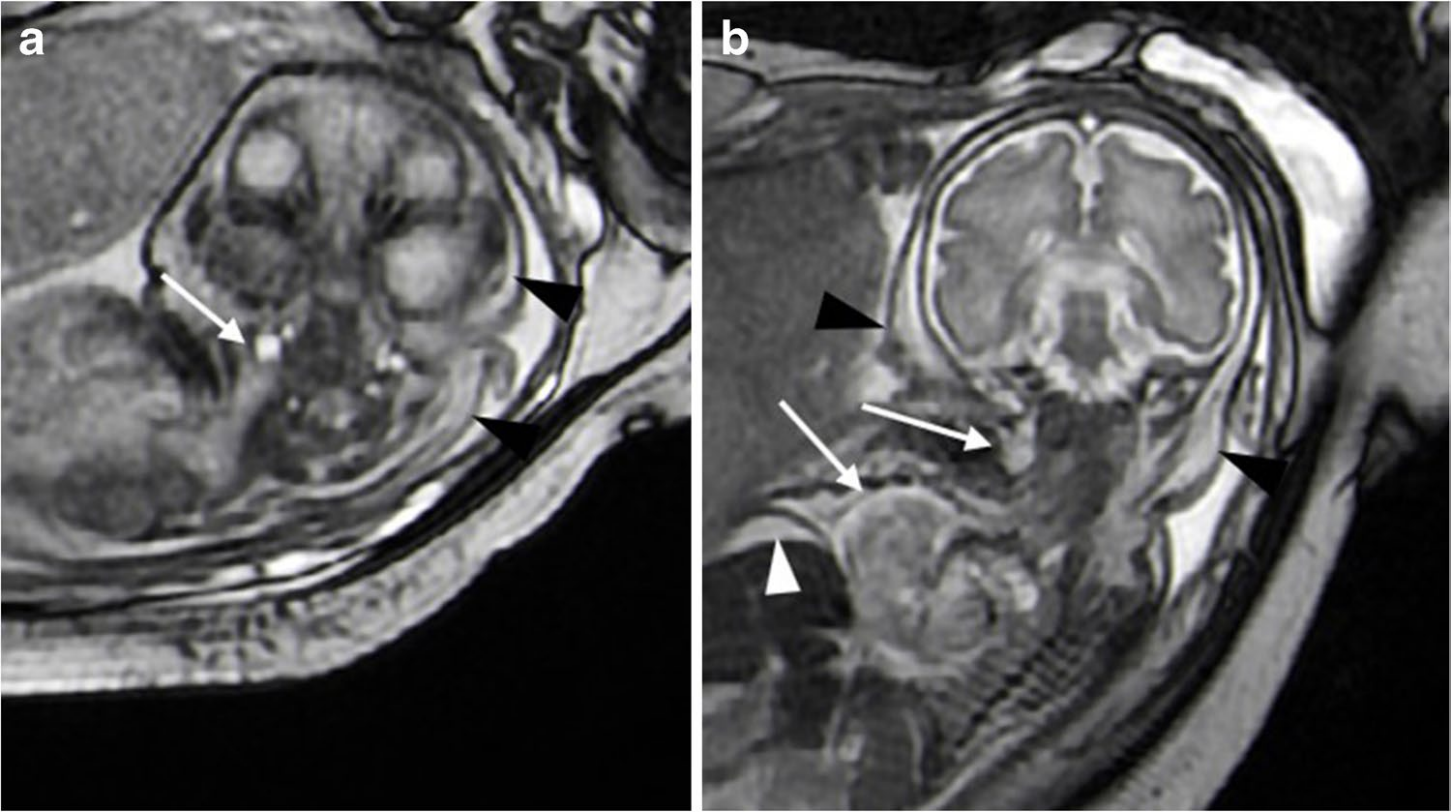
Magnetic resonance imaging in a 30-week-old male fetus with hydrops. **a** An axial balanced steady-state free precession (B-SSFP) image at the level of the atlantoaxial joint shows fetal scalp oedema (*arrowheads*) and internal jugular vein dilatation (*arrow*), **b** A coronal B-SSFP image shows fetal scalp thickening due to oedema (*black* *arrowhead*), ascites (*white* *arrowhead*), right atrial enlargement and right internal jugular vein dilatation (*arrows*)

Magnetic resonance imaging of another patient, a 30-week-old male fetus (Fig. 7), showed diffuse skin swelling and thickening, ascites, enlargement of the right heart and dilatation of the right internal jugular vein.

## Discussion

The internal jugular veins are connected to the sigmoid sinus and exit the skull through the base of the jugular foramen. The internal jugular veins collect blood from the brain, face and part of the scalp, merging with the subclavian vein downwards to the brachiocephalic vein and then return blood to the right atrium through the superior vena cava. The internal jugular veins may be affected in a variety of intracranial conditions. Imaging studies of the internal jugular veins have shown that abnormal development of the internal jugular veins in adult patients often leads to pulsatile tinnitus and vertigo [18, 19]. In children, some diseases, such as dural arteriovenous fistula and achondroplasia, can cause changes in blood flow and morphology of the internal jugular veins. Similarly, abnormality of the fetal internal jugular veins is also associated with a variety of diseases. In fetuses with intrauterine growth restriction, cerebral and systemic blood flow is redistributed, while the cerebral venous system is dilated [12, 20]. In contrast to the ability of Doppler ultrasound to detect blood flow changes, MRI can be used to observe the morphological changes in fetal vessels. Both B-SSFP and SS-FSE sequences can show anatomical structure and abnormal lesions of the fetal organs. This study showed that the B-SSFP sequence provides clear images of the fetal internal jugular veins with high signal intensity, making vascular anatomy more conspicuous compared to adjacent internal carotid artery, muscles and solid organs. Therefore, the B-SSFP sequence has utility for imaging and measurement of the fetal internal jugular veins. The internal jugular veins and surrounding tissues both showed low signal and indistinct boundaries on the SS-FSE sequence. Because of the low resolution, DWI and FIRM sequences are not considered suitable for imaging of the fetal internal jugular veins.

In this study, we chose to measure the fetal internal jugular veins at the level of the atlantoaxial joint for two reasons. First, excessive stretching or bending in some fetal positions will lead to internal jugular vein deformation with potential measurement errors. However, at the level of the atlantoaxial joint, the structures around the internal jugular veins are limited and there is only a small range of motion, so the shape of the internal jugular vein lumen is minimally affected. Furthermore, the internal jugular veins at the level of the atlantoaxial joint are adjacent to the skull base and mainly receive blood from the cerebral vein system, allowing them to more accurately reflect the changes in cerebral blood flow [21]. The results of the present study indicate that the normal cross-sectional area of fetal internal jugular veins is positively correlated with gestational age in the middle and third trimesters, suggesting that with the increase in fetal brain volume and blood flow supply, the venous return flow also increases. The cross-sectional area of the right internal jugular vein is larger than that of the left in fetuses, especially in late pregnancy, which is consistent with findings in most in adults. The difference in gene expression and transcription determines the existence of the dominant hemisphere of the brain, which is not affected by age or sex, and this phenomenon has been observed in early pregnancy [22, 23]. The internal jugular veins mainly receive venous blood returning from the sigmoid sinus and the larger cross-sectional area of the right internal jugular vein may be the continuation effect of this developmental advantage. However, no obvious right-sided dominance of the jugular foramen was found in anatomical observations of the fetal skull base [24]. It is possible that the wall of the internal jugular veins is elastic and can easily expand, however, the jugular foramen is a bony structure and difficult to change.

In addition, this study revealed that the cross section of fetal internal jugular veins was mostly round, but the proportion with an oval shape increased in the late gestational age group. The possible reason is that with the development of the fetus, adjacent structures gradually grow and squeeze the internal jugular veins [25]. Given the thin and high elastic venous wall, these morphological changes are reasonable.

Some internal jugular vein anomalies were included in this study. Cerebral arteriovenous fistula, VGAM, fetal hydrops, intrauterine fetal growth restriction and other diseases, may cause unilateral or bilateral internal jugular vein dilatation. The normal reference values can be used to evaluate the degree of internal jugular vein dilatation. The degree of internal jugular vein dilatation may be related to fetal prognosis and could guide clinical practice. Some fetuses with congenital diseases such as congenital diaphragmatic hernia, need surgical treatment in the neonatal period. Fetal MRI is often performed before surgery to evaluate the cardiothoracic ratio and the content of hernias. Development of internal jugular veins should be assessed to prepare for the central vein catheterisation required for extracorporeal membrane oxygenation (ECMO). Dysplasia of fetal internal jugular veins is one of the main reasons for ECMO installation failure, due to growth restriction [26]. Magnetic resonance imaging is needed for the assessment of neck vascular anatomy of the fetus with congential diaphragmantic hernia, particularly during late pregnancy, at which time vessels will be expected to be proportionately larger and more conspicuous and present fewer artefacts from fetal motion. Right heart failure may occur in some fetuses with hydrops fetalis, resulting in increased pressure of returning blood flow and secondary dilation of the outflow veins. Dilation of the internal jugular veins may indicate obstruction of cerebral venous return.

This study has some limitations that need to be considered. First, the sample size was small, especially with regard to fetuses under 28 and above 38 weeks of gestation. The sample size  needs to be increased in future studies. Second, a combination of blood flow as measured by ultrasound and morphological observation by MRI is optimal for identifying any lesions associated with abnormalities of the fetal internal jugular veins. Third, the degree of dilatation of the internal jugular veins may be related to prognosis, which should be studied further.

## Conclusion

In conclusion, the morphological MRI data obtained in this study may provide a basis for the clinical assessment of abnormal dilatation, or (to some extent) stenosis of the fetal internal jugular veins.

## Supplementary Information

Below is the link to the electronic supplementary material.

Supplementary file1 (DOCX 27 kb)

## Data Availability

The data used to support the findings of this study are available from the corresponding author upon request.
