# Supplementary material for: Normal reference values for magnetic resonance imaging measurements of the fetal internal jugular veins in middle and late pregnancy
Source: Pediatr Radiol. 2023 Mar 28;53(5):920–8. doi: 10.1007/s00247-023-05594-w (PMC10156768; doi:10.1007/s00247-023-05594-w)
Supplement: Supplementary file 1 — Supplementary file1 (DOCX 27 kb) [file 247_2023_5594_MOESM1_ESM.docx]

The flowchart with the number of patients inclusion and exclusion.

Middle and late pregnancy who underwent MRI examination in the Second Hospital of Hebei Medical University from October 2019 and July 2020. (n=179)

Pregnant women < 18 years old (n=2);

Pregnant woman with twins (n=1)

Single fetuses in middle and late pregnancy (n=176)

Poor image quality (n=19);

The B-SSFP sequence was missing(n=3)

Single fetuses in middle and late pregnancy with normal internal jugular veins (n=126)

Single fetuses in middle and late pregnancy with abnormal internal jugular veins (n=28)

GVAM (n=10);

Leptoleningeal arteriovenous fistula (n=5);

Fetal edema (n=10);

Intrauterine fetal growth restriction (n=3)
